# Cardiac response to water activities in children with Long QT syndrome type 1

**DOI:** 10.1371/journal.pone.0295431

**Published:** 2023-12-07

**Authors:** Anna Lundström, Urban Wiklund, Annika Winbo, Håkan Eliasson, Marcus Karlsson, Annika Rydberg

**Affiliations:** 1 Department of Clinical Sciences, Pediatrics, Umeå University, Umeå, Sweden; 2 Department of Radiation Sciences, Radiation Physics, Biomedical Engineering, Umeå University, Umeå, Sweden; 3 Department of Physiology, University of Auckland, Auckland, New Zealand; 4 Department of Women’s and Children’s Health, Karolinska Institutet, Stockholm, Sweden; University of Tampere, FINLAND

## Abstract

**Background:**

Swimming is a genotype-specific trigger in long QT syndrome type 1 (LQT1).

**Objective:**

To examine the autonomic response to water activities in children and adolescents with LQT1.

**Methods:**

In this cross-sectional study, LQT1 patients were age and sex matched to one healthy control subject. Electrocardiograms (ECGs) were recorded during face immersion (FI), swimming, diving, and whole-body submersion (WBS). Heart rate (HR) and heart rate variability (HRV) was measured. The high frequency (HF) component of HRV was interpreted to reflect parasympathetic activity, while the low frequency (LF) component was interpreted as reflecting the combined influence of sympathetic and parasympathetic activity on autonomic nervous modulation of the heart.

**Results:**

Fifteen LQT1 patients (aged 7–19 years, all on beta-blocker therapy) and fifteen age and sex matched non-medicated controls were included. No significant ventricular arrhythmias were observed in the LQT1 population during the water activities. Out of these 15 matched pairs, 12 pairs managed to complete FI and WBS for more than 10 seconds and were subsequently included in HR and HRV analyses. In response to FI, the LQT1 group experienced a drop in HR of 48 bpm, compared to 67 bpm in the control group (p = 0.006). In response to WBS, HR decreased by 48 bpm in the LQT1 group and 70 bpm in the control group (p = 0.007). A significantly lower PTOT (p < 0.001) and HF (p = 0.011) component was observed before, during and after FI in LQT1 patients compared with the controls. Before, during and after WBS, a significantly lower total power (p < 0.001), LF (p = 0.002) and HF (p = 0.006) component was observed in the LQT1 patients.

**Conclusion:**

A significantly lower HR decrease in response to water activities was observed in LQT1 subjects on beta-blocker therapy, compared to matched non-medicated controls. The data suggests an impaired parasympathetic response in LQT1 children and adolescents. An aberrant autonomic nervous system (ANS) response may cause an autonomic imbalance in this patient group.

## Introduction

Long QT syndrome (LQTS) is primarily caused by pathogenic variants in three main genes: KCNQ1, KCNH2, and SCN5A. These genes encode cardiac ion channel subunits, corresponding to the LQTS subtypes LQT1, LQT2, and LQT3, respectively [1]. All LQTS subtypes are associated with arrhythmia-induced syncope and risk of sudden cardiac death (SCD). However, the clinical phenotypes and triggers that causes ventricular arrhythmias differ depending on the LQTS subtype [2]. For LQT1, the main triggers are physical activity, emotional stress, and swimming, which are related to changes in sympathetic autonomic tone [3]. Beta-blockers, which act on the sympathetic branch of the autonomic nervous system (ANS), are the mainstay therapeutic option in LQTS, and are highly effective in LQT1 [3].

Swimming appears to carry the highest risk of exercise-related SCD in individuals with LQT1, therefore it is reasonable to impose restrictions not only in competitive settings, but also for recreational swimming [4]. Furthermore, the risk appears to be even more pronounced during adolescence [5]. As there are currently few studies regarding cardiac response during water activities in children and adolescents with LQTS, this constitutes a clear knowledge gap.

Unlike many other stimuli, water activities have the potential to activate both branches of the ANS simultaneously [6]. The sympathetic branch is activated following the cold shock response or due to adrenaline released during water activities. This results in an increased heart rate (HR) and peripheral vasoconstriction, diverting oxygenated blood to vital organs. When submerging under water with concomitant breath-holding, the oxygen-preserving diving reflex becomes activated, leading to parasympathetic activation via the vagus nerve and consequently decreasing HR [7]. This conflicting dual ANS activation has previously been proposed as a significant contributor to why swimming acts as such a strong trigger for life-threatening cardiac events in LQT1 [3].

Due to technical improvements, it is now possible to monitor the HR during water activities with a small portable electrocardiogram (ECG) recorder. The recorder allows for the assessment of the presence of arrhythmias and the investigation of the autonomic response by measuring the heart´s beat-to-beat adjustment in HR, i.e., the heart rate variability (HRV). This standardized method enables estimation of ANS activity during submersion [8]. The aim of this study was to assess the presence of arrhythmias and to examine the autonomic response to water activities in children and adolescents with LQT1 compared to healthy controls.

## Materials and methods

### Study population

Seventeen LQT1 children and adolescents aged 6 to 19 years, who attended regular cardiology follow-up at the Department of Pediatric Cardiology, Umeå University Hospital, Sweden, were invited to participate. The LQT1 diagnosis had been genetically confirmed in all patients, and the genetic variants were classified according to the American College of Medical Genetics (ACMG) guidelines (S1 Table) [9]. Recruitment and data collection occurred between 2019 and 2022. Data regarding previous symptoms, ongoing beta-blocker therapy, therapy compliance, other medical conditions, additional medications, and intake of nicotine or caffeine were noted. A control group was recruited from the same region. Each LQT1 patient was matched with a healthy control of the same age and sex. None of the controls were on beta-blocker therapy. Informed written consent was obtained from all participants’ legal guardians. Defibrillator and personnel trained in cardiopulmonary resuscitation (CPR) were on-site during all water activities. The study was approved by the Regional Ethical Review Board (Umeå University, Dnr 05–127, Dnr 05-127M) and is in concordance with the Declaration of Helsinki.

### Swimming protocol

All water activities and recordings were performed indoors, with air temperature between 27-28°C, during the afternoon. All participants followed a protocol (S2 Table) consisting of five water activities (events) with 3 minutes rest (sitting and without speaking) before, in between and after each event: 1) face immersion (FI) in 10°C water for up to 25 seconds; 2) whole-body submersion (WBS) in 27°C water for up to 25 seconds; 3) swimming with the head above water for 25 meters; 4) diving head-first (if possible depending on the child’s swimming skills); and 5) diving head-first and then continuing swimming under water for the duration of at least 5 swim strokes. During all five events the presence of significant arrhythmias was assessed using recordings from ECG monitoring equipment. We additionally assessed the presence of ECG changes such as T-wave alternans and short-long-short sequences which have been shown to be precursors to Torsades de Points [10, 11]. Events 1 and 2 were also performed with the purpose of studying the cardiac response during the diving reflex. Only subjects with >10 seconds duration for each event were included in the analyses. This analysis was not performed on the recordings from events 3, 4 and 5, as the extensive activation of the pectoral muscles during swimming significantly increased disturbances in the ECG recordings.

### Heart rate monitoring

The Actiwave-Cardio monitor (CamNtech, Cambridge, UK) was used to continuously record the ECG signal for all subjects. The device is a waterproof single-channel ECG recorder with a built-in accelerometer. Both the device and electrodes were covered with water-resistant adhesive plasters, which significantly reduced the disturbances noted in recordings made without the cover, as observed in a pilot study. The onset and end of each event were determined based on times noted in the protocol. However, these time points were adjusted after visual inspection of changes in the 3-axis acceleration signals during raw data post-processing. The ECG was sampled at 500 Hz and heart beats were detected using custom-made software. Errors in the detection were manually corrected, and the presence of arrhythmic beats were noted. The baseline HR was defined as the average of the last two heart beats before the start of each event. The maximum and minimum HR were determined during each event. However, if the HR pattern was consistent with a single premature atrial contraction that occurred at the onset or end of an event, and thereby resulted in a falsely high/low heart rate, this was manually corrected. The series of heartbeats from the period 40 seconds before to 60 seconds after each event were converted to equidistantly sampled data by cubic spline interpolation and resampling at 4.8 Hz. QTc duration in the LQT1 patients was manually calculated from a standard 12-lead resting ECG recorded in a clinical setting at the hospital. The QTc in the controls was automatically calculated during the first resting phase using a custom-made software. Bazett’s formula was used to correct QT time for HR [12].

### Heart rate variability

Heart rate variability (HRV) was analyzed in subjects who performed both the FI and WBS events for >10 seconds. Due to the short duration of the provocations, instead of using traditional methods for power spectrum analysis, the wavelet transform was applied to assess instantaneous changes in HRV [13]. The wavelet transform has previously been used to analyze HRV during FI in healthy subjects [14]. In short, the method used for extraction of spectral components from wavelet transformation [15] acts as a set of bandpass filters, where the fluctuations in low-frequency (LF, 0.04–0.15 Hz) and high-frequency (HF, 0.15–0.60 Hz) regions are extracted in the time-domain. The HF component mainly represents parasympathetic activity, whereas LF component represents the combined sympathetic and parasympathetic effect on cardiac autonomic nervous modulation. The LF/HF ratio was also calculated. HRV was assessed during three periods: 1) 20 seconds before the start of the event; 2) during the event; and 3) 20 seconds after the end of the event. For each analyzed segment, total power (PTOT), which reflects the overall autonomic activity, was determined based on the variance of the fluctuations in HR after the mean value had been removed. The power of the spectral components was estimated as the mean squared value of the decomposed LF and HF components. Spectral indices were log-transformed using base 10. The decomposition was performed with the discrete wavelet transform using the symlet-10 wavelet and 5 levels. All analyses were performed using Matlab R2022b (Mathworks Inc, Natick, MA, USA).

### Statistical analyses

The overall HR response was determined by calculating group averages and standard errors of the mean (SEM) values from the equidistantly sampled data. The responses were smoothed by calculating one-second moving averages. QTc was reported as median ± standard deviation (SD). Comparisons between the two groups regarding HR response, duration of the events, and QTc duration were conducted with two-sampled t-tests. Changes in HRV data over time were analyzed by analysis of variance (ANOVA) of repeated measurements, with time, group, and their interactions as variables. The normality of the residuals was verified by the Shapiro-Wilks test. A p-value of < 0.05 was considered statistically significant. Statistical analysis was performed using IBM SPSS Statistics v28 (IBM Corp., Armonk, NY, USA).

## Results

### Participants

**Fig 1:**
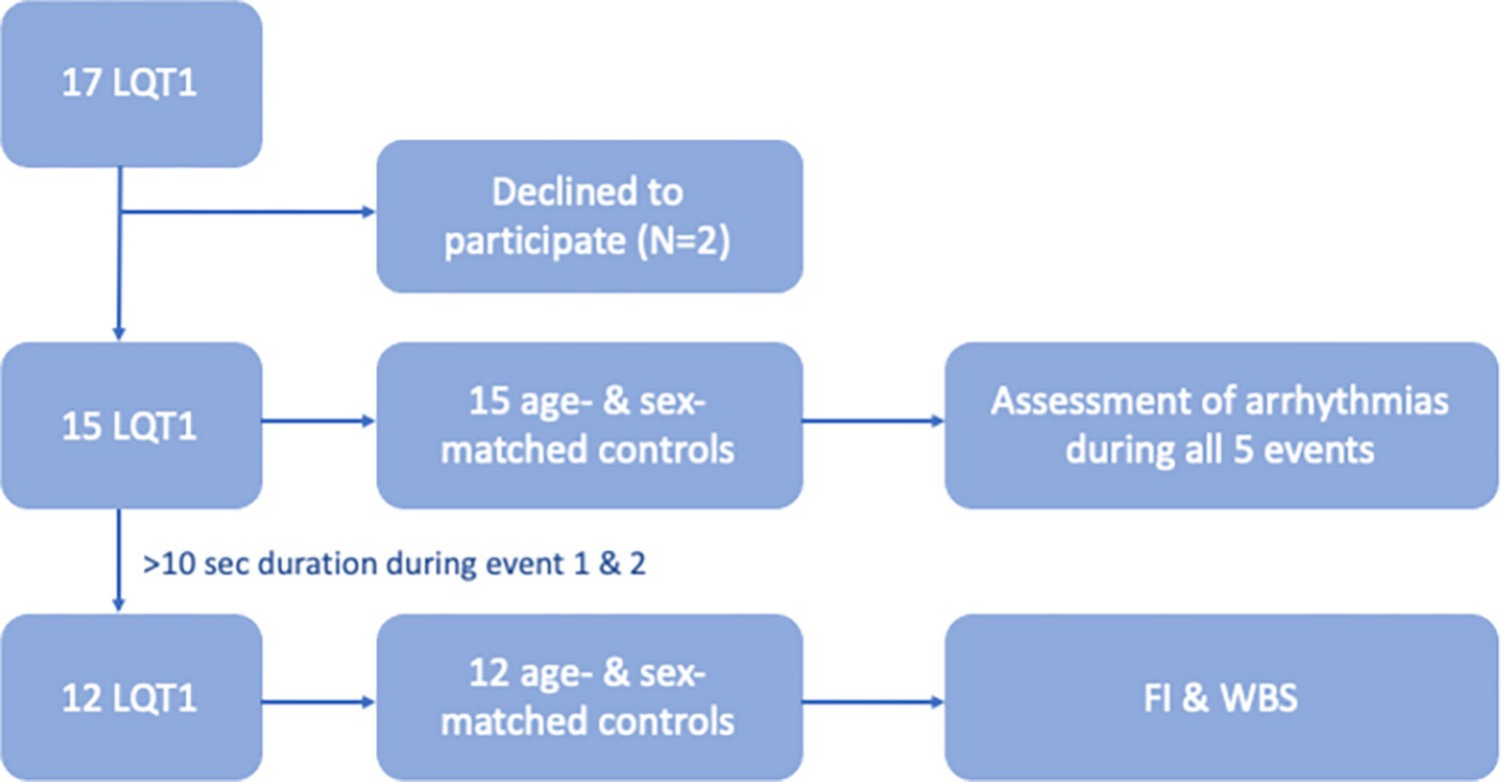
Flow chart of participant inclusion. LQT1 = long QT syndrome type 1, N = number, FI = face immersion, WBS = whole-body submersion.

Seventeen LQT1 children and adolescents were invited to participate in this study. Only two patients declined the invitation to participate (Fig 1). Consequently, a total of 15 LQT1 patients were included in the study, along with 15 age-and sex-matched healthy, medication-free controls. Among the 15 LQT1 patients, 9 were girls (60%, aged 7–19 years) and 6 were boys (40%, aged 7–12 years). Thirteen of the 15 patients had the Swedish founder mutation KCNQ1 Y111C. Median QTc in the LQTS group was 470 ± 22 ms, and in the control group 419 ± 22 ms (p < 0.001). Three patients had a history of syncope. Three siblings had a family history of near-drowning due to LQTS related events. All patients were on beta-blocker therapy, which was maintained during the study for ethical reasons. Beta-blockers are metabolized by the drug-metabolizing enzymes CYP2D6 and CYP2C19, and individuals’ genetic variation related to genetic polymorphism results in a wide range of metabolizing ability [16, 17]. Eight patients were on a low dose of beta-blocker medication due to being genetically diagnosed as poor metabolizers, while three had not been tested yet (Table 1). None of the subjects had an implantable cardioverter-defibrillator (ICD). Two LQTS patients and one control had asthma; however, none of these subjects had taken any medication. None of the participants had consumed any caffeine or nicotine containing products that could have affected the heart during 8 hours before the events in this study.

**Table 1:** Clinical characteristics of LQTS type 1 patients.

N	Sex	Age (years)	QTc (ms)	Syncope*	Beta-blocker	Dose** (mg/kg/day)
1	Male	7	490	No	Prop	1.0
2	Male	8	480	No	Met	0.7
3	Male	9	469	Yes	Met	0.4
4	Male	9	460	Yes	Met	0.5
5	Male	11	455	No	Met	0.7
6	Male	12	515	No	Met	0.6
7	Female	7	440	No	Prop	0.9
8	Female	9	460	No	Met	0.5
9	Female	10	490	No	Met	0.4
10	Female	10	470	No	Prop	1.3
11	Female	11	435	No	Prop	0.9
12	Female	14	470	No	Met	0.5
13	Female	17	480	No	Prop	1.2
14	Female	17	465	Yes	Met	0.5
15	Female	19	510	No	Met	0.5

QTc was manually calculated with Bazett’s formula from a standard 12-lead resting ECG. Genotype in all patients: LQT1. N = number, QTc = corrected QT time, Met = metoprolol, Prop = propranolol.

* History of arrhythmogenic syncope.

**Several patients had a low dose of beta-blocker therapy after being genetically diagnosed as poor metabolizers.

### Arrhythmias

Among the 15 LQT1 patients, no significant ventricular arrhythmias or T-wave alternans were observed during any of the five events. Although we noticed increased variability in the RR-length at the end of both FI and WBS in both controls and LQT1 patients, no short-long-short sequences could be identified. A 17-year-old female control was excluded due to recurrent ventricular bigeminy during water activities. She was referred for further examinations, and a new control was recruited.

### Heart rate

**Fig 2:**
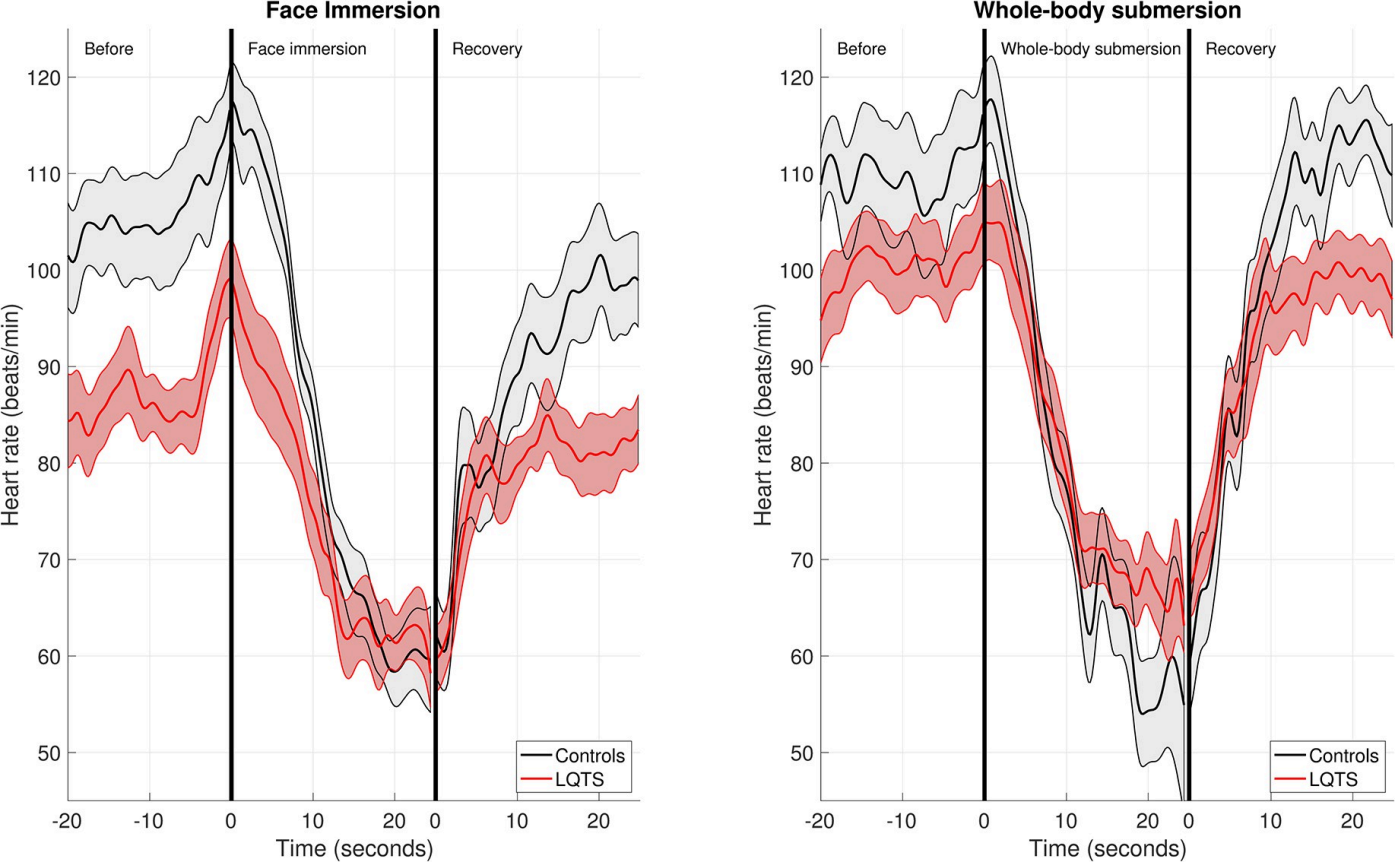
Heart rate changes during face immersion and whole-body submersion. Data is presented as means and standard error of the mean (SEM). N = 12 for both LQTS patients and controls. LQTS = long QT syndrome.

**Fig 3:**
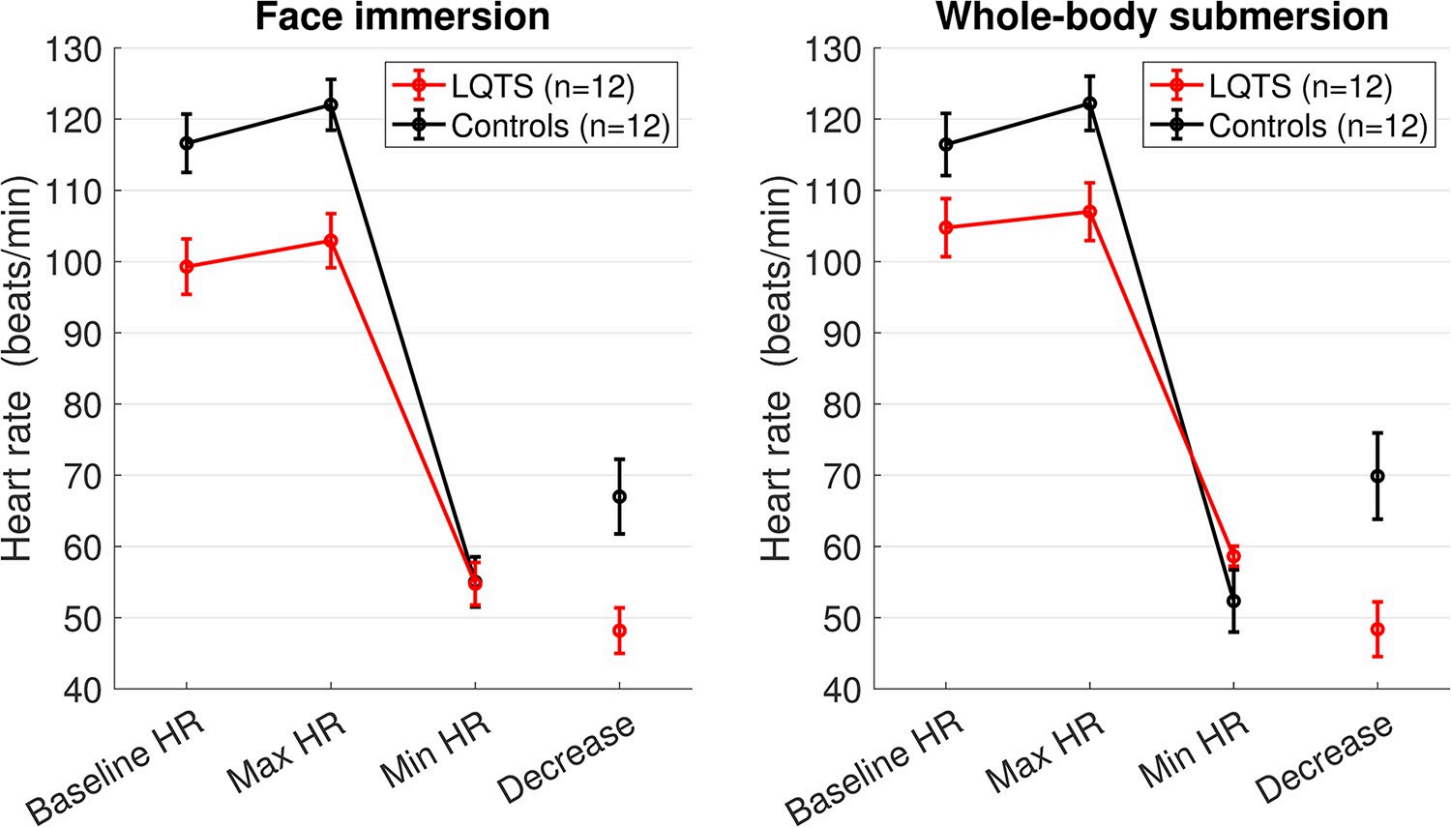
Maximal and minimal heart rate during each event compared to the heart rate at the start of each event (baseline). Data is presented as mean and SEM. Decrease = difference between Max and Min HR. LQTS = long QT syndrome, HR = heart rate.

Out of the 15 matched LQT1 patient and control pairs, 12 had a duration time >10 seconds for events 1 and 2 (FI and WBS) and were thus included in the HR and HRV analyses. As seen in Fig 2, all participants had an increase in HR just before the start of each event. At the start of the FI, the HR at baseline was 99 bpm for the LQT1 patients and 117bpm for the controls (p = 0.006). A lower initial HR was expected in the LQT1 group due to beta-blocker therapy (Fig 3 and Table 2). In response to FI, the LQT1 group experienced a drop in HR of 48 bpm, compared to 67 bpm in the control group (p = 0.006). At the start of WBS, the HR at baseline was 105 bpm for the LQT1 patients and 116 bpm for the controls (p = 0.06). In response to WBS, the HR decreased by 48 bpm in the LQT1 group and 70 bpm in the control group (p = 0.007).

**Table 2:** Heart rate during face immersion and whole-body submersion in LQT1 patients and controls.

**Face immersion**	Controls (N = 12)	LQT1 (N = 12)	p-value
Baseline HR	117 (14)	99 (14)	0.006*
Max HR	122 (12)	103 (13)	0.001*
Min HR	55 (12)	55 (10)	0.96
Decrease	67 (18)	48 (11)	0.006*
Decrease%	54 (11)	47 (8)	0.06
Duration (sec)	22.2 (4.4)	22.3 (5.2)	0.97
**Whole-body submersion**	Controls	LQT1	p-value
Baseline HR	116 (15)	105 (14)	0.06
Max HR	122 (13)	107 (14)	0.01*
Min HR	52 (15)	59 (5)	0.18
Decrease	70 (21)	48 (13)	0.007*
Decrease%	57 (13)	44 (8)	0.01*
Duration (sec)	20.4 (5.8)	21.7 (5.7)	0.59

Values are given as beats per minute, duration in seconds or as %. Data are presented as mean (SD). P-values are from two-sided t-tests.

* p < 0.05. LQT1 = long QT syndrome type 1, HR = heart rate.

### Heart rate variability

Table 3 shows the data from the HRV analysis in LQT1 patients and controls. There was a significantly lower PTOT (p < 0.001) and HF (p = 0.011) before, during and after FI in LQT1 patients compared with the controls. During FI, the LQT1 patients had a significantly higher LF/HF ratio than controls (p = 0.018). Before, during and after the WBS, LQT1 patients also presented with a significantly lower PTOT (p < 0.001), LF (p = 0.002) and HF (p = 0.006).

**Table 3:** Heart rate variability before, during and after face immersion and whole-body submersion in LQT1 patients and controls.

**Face immersion**	Controls (N = 12)			LQT1 (N = 12)			ANOVA p-values		
	Before	During	Recovery	Before	During	Recovery	Phase	Grp	Grp x Phase
PTOT	1.89 (0.30)	2.57 (0.33)	2.26 (0.22)	1.77 (0.25)	2.18 (0.31)	1.88 (0.16)	<0.001*	<0.001*	0.12
LF	0.99 (0.50)	0.85 (0.40)	1.29 (0.33)	0.83 (0.48)	0.85 (0.44)	1.05 (0.35)	0.012*	0.27	0.56
HF	0.76 (0.52)	0.80 (0.55)	1.14 (0.37)	0.52 (0.40)	0.19 (0.55)	0.85 (0.45)	<0.001*	0.011*	0.29
LF/HF	0.24 (0.29)	0.06 (0.47)	0.15 (0.28)	0.31 (0.44)	0.65 (0.40)	0.20 (0.23)	0.20	0.018*	0.013*
HRmean (beats/min)	106.47 (16.45)	87.74 (9.31)	88.61 (14.59)	87.74 (11.16)	76.81 (14.48)	78.86 (10.73)	<0.001*	0.006*	0.21
**Whole-body submersion**	Controls			LQT1			ANOVA p-values		
	Before	During	Recovery	Before	During	Recovery	Phase	Grp	Grp x Phase
PTOT	1.86 (0.34)	2.61 (0.31)	2.50 (0.34)	1.51 (0.29)	2.33 (0.37)	2.18 (0.23)	<0.001*	<0.001*	0.94
LF	1.02 (0.57)	1.29 (0.42)	1.06 (0.53)	0.51 (0.47)	0.70 (0.60)	0.81 (0.55)	0.28	0.002*	0.51
HF	1.12 (0.32)	1.08 (0.41)	1.26 (0.62)	0.58 (0.49)	0.57 (0.44)	1.06 (0.57)	0.020	0.006*	0.33
LF/HF	-0.10 (0.35)	0.21 (0.26)	-0.20 (0.70)	-0.07 (0.39)	0.13 (0.48)	-0.25 (0.40)	0.012*	0.76	0.88
HRmean (beats/min)	110.13 (17.64)	84.27 (10.32)	98.03 (14.88)	100.70 (12.05)	83.22 (10.93)	92.92 (10.54)	<0.001*	0.25	0.27

Data are presented as mean (SD). Values are log-10 transformed HRV expressed in (beats/min)^2^.

* p < 0.05 for LQTS vs controls. LQT1 = Long QT syndrome type 1, Grp = group, PTOT = total power, LF = power of the low frequency component, HF = power of the high frequency component, LF/HF = the ratio between the low frequency component and the high frequency component, HRmean = mean heart rate.

## Discussion

In this study we have assessed the presence of arrhythmias and the autonomic response to water activities in children and adolescents with LQT1 while on their individual regular beta-blocker therapy. None of the included LQT1 patients had malignant arrhythmias during the water activities. However, a less pronounced HR decrease was observed in LQT1 patients, compared to healthy age- and sex-matched controls. Furthermore, during FI and WBS, the LQT1 patients had a decreased HRV, especially in the HF spectrum, indicating reduced parasympathetic activity. These findings suggest differences between LQT1 patients and controls in autonomic response and imply that the diving reflex provoked a less pronounced parasympathetic activation in the LQT1 patients.

Water activities are associated with arrhythmias, even in healthy individuals [18, 19]. Since ECG monitoring has historically been challenging to perform in water, previous studies on the diving reflex have often been executed in a more controlled setting on land, and FI has been shown to be a reliable substitute [6]. Ishikawa et al. demonstrated that arrhythmias were highly inducible during FI and WBS in children with a previously known tendency to develop ventricular tachycardia or ventricular premature contractions [20]. In our study, none of the LQT1 patients on beta-blocker therapy developed ventricular arrhythmias. However, one of the healthy controls developed recurrent ventricular bigeminy during water activities and was subsequently excluded. This occurrence highlights the increased arrhythmia risk even in seemingly healthy individuals during water activities.

Water activities have been known to be a particularly malignant trigger in LQTS patients, especially during childhood [21]. The 2022 ESC Guidelines for the management of patients with ventricular arrhythmias and the prevention of SCD recommend that individuals with LQT1 should avoid strenuous swimming [22]. Furthermore, the ESC guidelines on sports cardiology and exercise in patients with cardiovascular disease (2020), conclude that LQT1 patients should not engage in sports that involve diving into cold water [23]. In a study by Choi et al., among subjects referred for LQTS genetic testing, 43 of 388 (11%) had a personal and/or family history of a near-drowning or drowning [24]. Of these 43 cases, 28 were determined to have a LQT1 genotype. This highlights the importance of studying swimming in LQT1 patients since water activities, unlike many other stimuli, can simultaneously activate both branches of the ANS, potentially contributing to their heightened risk of life-threatening cardiac events [6].

In 2002, Batra et al. published a case report documenting a 12-year-old female with a prolonged QT interval and an ICD, who experienced ventricular tachycardia and Torsades de pointes (TdP) while swimming [25]. Interestingly, the tachycardia was proceeded by marked variability in both rate and QRS morphology, followed by a full compensatory pause of 0.70 seconds. Furthermore, the same pattern could be seen in another case study involving a 12-year-old male with LQT2, who experienced TdP during FI in 10-degree water [26]. The sudden cold-water immersion seemed to cause an autonomic imbalance that eventually triggered the TdP.

In 2012, the theory of ‘autonomic conflict’ was introduced by Shattock and Tipton, suggesting that cold water immersion simultaneously activates two strong autonomic reflexes; the diving reflex and the cold shock response [27]. Apnea and the sensation of water on the face and nose activate the parasympathetic diving reflex via the vagal nerve, leading to bradycardia and reduced workload on the heart, resulting in reduced oxygen consumption [27]. The sympathetically driven cold shock response, activated through cutaneous cold thermoreceptors, causes the direct opposite reaction, with tachycardia, hyperventilation, peripheral vasoconstriction, and hypertension [27].

In 2014, Shamsuzzaman et al. demonstrated that simulated diving leads to strong sympathetic activation [6]. Also, in a recent study from our research group on human induced pluripotent stem cell-derived sympathetic neurons from LQT1 patients, a significant sympathetic neuronal hyperactivity was identified at baseline [28]. Unfortunately, a sympathetic assessment could not be conducted in this current study due to the short duration of the events, the presence of beta-blocker therapy in the LQT1 patients, and the spectral components LF and PTOT representing a combination of both branches of the ANS. With that caveat stated, we were mainly able to assess the parasympathetic response to water activities. Our findings, showing a decreased HF and increased LF/HF during FI, and an overall decrease in HRV during WBS, indicate that the parasympathetic branch of the ANS appears less active in children with LQT1. Theoretically, a combination of sympathetic hyperactivity and depressed parasympathetic activity could result in a significant autonomic imbalance, which might contribute to arrhythmogenesis in these patients.

Our findings of a lower reduction in HR during FI in LQT1 patients are consistent with those of Yoshinaga et al. [29]. In their study, which included 17 children with prolonged QT interval, the high probability LQTS group showed a 43% reduction in HR, whereas the controls had a 50% reduction in HR during FI. Recently, Marstrand et al. published a study on adult LQTS patients [30]. They investigated the response to FI in 9 LQT1 and 14 LQT2 patients, both on and off beta-blocker therapy. The nine LQT1 patients showed a decrease in their HR by 35% while off beta-blocker therapy, and 40% while on beta-blocker therapy. In comparison, the controls showed a decrease in HR by 39%. It is well-known that beta-blockers have an impact on the sinus node, resulting in a lowered HR. However, it has also been shown that a selective beta-blocker can increase HRV in healthy subjects, particularly enhancing vagal tone [31]. In patients with hypertension, coronary heart disease or after a myocardial infarction, the effect of metoprolol on HRV parameters has been inconsistently reported, with metoprolol either having no effect or increasing HRV parameters [32–34]. In contrast, the beta-blocked LQTS patients in our study had a lower HF, indicating reduced vagal tone, compared to unmedicated healthy controls.

Previous studies have shown progressive increase in cardiac parasympathetic activity during early-to-late childhood [35]. However, a study from 2021 revealed a plateau phase in parasympathetic activity during middle childhood, and no change or a small decrease at the end of adolescence [36]. The same study concluded that it is notable that interindividual variation can be significant at all ages. In our study, the parasympathetic activity was investigated by comparing LQT1 subjects with age-and sex-matched healthy controls. This study design is suitable for comparing small groups but does not reduce the possible effect of interindividual variation. Further and larger studies are warranted to examine normal cardiac autonomic control and interindividual variations in individuals of all ages in childhood and adolescence.

Consistent with the findings of this study, a prior study conducted by our research group also observed an apparent reduction in parasympathetic activity in LQT1 patients [37]. In this previous study, we investigated the ANS response during the post-exercise phase in LQTS patients, both on and off beta-blockers, and found a depressed parasympathetic effect on both heart rate recovery (HRR) and HRV after exercise. DeMaria et al. reported similar results in a study of Holter recordings in adult LQTS patients [38]. The authors demonstrated that, across multiple physiological states, the total autonomic and vagal functions were lower in the LQTS group. Furthermore, they identified vagal function as an independent marker to distinguish between LQT1 and LQT2 patients and noted that symptomatic LQT1 patients demonstrated a reduced ANS function [38]. In this current study, HRV analysis showed a lower PTOT, LF and HF during all phases of the WBS, and a lower PTOT and HF during FI in the LQT1 children compared with the controls. This implies a difference in autonomic response, and the decreased HF in the LQT1 group during FI and WBS specifically indicates a lower parasympathetic activation.

That the parasympathetic response of the ANS in LQT1 patients deviates from that of the healthy controls is very interesting, especially considering findings in a study from Winter et al., which was conducted on rabbits with LQTS. This study demonstrated that the dynamic interplay between the two branches of the ANS is essential in the arrhythmogenesis in LQTS [39]. Although both the sympathetic and parasympathetic input to the heart can independently trigger arrhythmias, the alterations in the balance between them can even more strongly predispose for ventricular arrhythmias. This makes the theory of autonomic conflict more likely.

### Limitations

Our study included a small patient cohort, primarily attributed to the rarity of LQT1 and the inclusion of a 6 to 19-year age range. This may limit the ability to detect minor differences between the groups as it reduces the power of statistical analysis. Also, thirteen of the 15 patients had the mutation KCNQ1 Y111C corresponding to a relatively benign phenotype, which may possibly affect the results.

All our LQT1 patients were on beta-blocker therapy, as therapy discontinuation was not feasible from an ethical point of view. Also, initiating beta-blocker therapy in healthy children was also deemed ethically inappropriate. However, beta-blocker therapy is known to either enhance HRV parameters or have no impact. Interestingly, in this study, beta-blocked LQT1 patients consistently exhibited lower HRV, which contradicts the usual expectations. This strengthens our observation of reduced vagal tone in beta-blocked LQTS patients.

The short duration of recordings limited our ability to assess the sympathetic response to water activities. Additionally, it’s important to note that LF power represents both sympathetic and parasympathetic activity. Therefore, the sympathetic response was not entirely captured.

## Conclusion

The results of this study indicate an impaired parasympathetic response to FI and WBS in children with LQTS type 1 when compared to healthy controls. This aberrant ANS response may cause an autonomic imbalance. Additionally, our findings align with prior research that has demonstrated a reduced parasympathetic activity in LQTS patients. Furthermore, these results provide insight that alterations in autonomic tone might contribute to the arrhythmogenesis in LQTS, emphasizing the importance of studying both branches of the ANS in future studies.

## Supporting information

S1 Checklist STROBE statement—checklist of items that should be included in reports of observational studies. (DOCX)

S1 Table Genetic variants found in the LQTS group. (DOCX)

S2 Table Swimming protocol. (DOCX)

## References

[1] Adler A, Novelli V, Amin AS, Abiusi E, Care M, Nannenberg EA, et al. An International, Multicentered, Evidence-Based Reappraisal of Genes Reported to Cause Congenital Long QT Syndrome. Circulation. 2020;141(6):418–28. Epub 2020/01/28. doi: 10.1161/CIRCULATIONAHA.119.043132 ; PubMed Central PMCID: PMC7017940. 31983240 PMC7017940

[2] Schwartz PJ, Priori SG, Spazzolini C, Moss AJ, Vincent GM, Napolitano C, et al. Genotype-Phenotype Correlation in the Long-QT Syndrome: Gene-Specific Triggers for Life-Threatening Arrhythmias. Circulation. 2001;103(1):89–95. doi: 10.1161/01.cir.103.1.89 11136691

[3] Winbo A, Paterson DJ. The Brain-Heart Connection in Sympathetically Triggered Inherited Arrhythmia Syndromes. Heart Lung Circ. 2020;29(4):529–37. Epub 20191216. doi: 10.1016/j.hlc.2019.11.002 . 31959550

[4] Christou GA, Vlahos AP, Christou KA, Mantzoukas S, Drougias CA, Christodoulou DK. Prolonged QT Interval in Athletes: Distinguishing between Pathology and Physiology. Cardiology. 2022;147(5–6):578–86. Epub 20220810. doi: 10.1159/000526385 . 35947943

[5] Albertella L, Crawford J, Skinner JR. Presentation and outcome of water-related events in children with long QT syndrome. Arch Dis Child. 2011;96(8):704–7. Epub 20101203. doi: 10.1136/adc.2009.178152 . 21131640

[6] Shamsuzzaman A, Ackerman MJ, Kuniyoshi FS, Accurso V, Davison D, Amin RS, et al. Sympathetic nerve activity and simulated diving in healthy humans. Auton Neurosci. 2014;181:74–8. doi: 10.1016/j.autneu.2013.12.001 ; PubMed Central PMCID: PMC4249686. 24368150 PMC4249686

[7] Panneton WM. The mammalian diving response: an enigmatic reflex to preserve life? Physiology (Bethesda). 2013;28(5):284–97. Epub 2013/09/03. doi: 10.1152/physiol.00020.2013 ; PubMed Central PMCID: PMC3768097. 23997188 PMC3768097

[8] Malik M, Bigger JT, Camm AJ, Kleiger RE, Malliani A, Moss AJ, et al. Heart rate variability: Standards of measurement, physiological interpretation, and clinical use. European Heart Journal. 1996;17(3):354–81. doi: 10.1093/oxfordjournals.eurheartj.a014868 8737210

[9] Richards S, Aziz N, Bale S, Bick D, Das S, Gastier-Foster J, et al. Standards and guidelines for the interpretation of sequence variants: a joint consensus recommendation of the American College of Medical Genetics and Genomics and the Association for Molecular Pathology. Genet Med. 2015;17(5):405–24. Epub 20150305. doi: 10.1038/gim.2015.30 ; PubMed Central PMCID: PMC4544753. 25741868 PMC4544753

[10] Malfatto G, Beria G, Sala S, Bonazzi O, Schwartz PJ. Quantitative analysis of T wave abnormalities and their prognostic implications in the idiopathic long QT syndrome. Journal of the American College of Cardiology. 1994;23(2):296–301. doi: 10.1016/0735-1097(94)90410-3 7905012

[11] El-Sherif N, Caref EB, Chinushi M, Restivo M. Mechanism of arrhythmogenicity of the short-long cardiac sequence that precedes ventricular tachyarrhythmias in the long QT syndrome. J Am Coll Cardiol. 1999;33(5):1415–23. doi: 10.1016/s0735-1097(98)00700-1 . 10193747

[12] Bazett HC. An analysis of the time-relations of electrocardiograms. Annals of Noninvasive Electrocardiology. 1997;2(2):177–94. 10.1111/j.1542-474X.1997.tb00325.x.

[13] Pichot V, Gaspoz JM, Molliex S, Antoniadis A, Busso T, Roche F, et al. Wavelet transform to quantify heart rate variability and to assess its instantaneous changes. J Appl Physiol (1985). 1999;86(3):1081–91. doi: 10.1152/jappl.1999.86.3.1081 . 10066727

[14] Kinoshita T, Nagata S, Baba R, Kohmoto T, Iwagaki S. Cold-water face immersion per se elicits cardiac parasympathetic activity. Circ J. 2006;70(6):773–6. doi: 10.1253/circj.70.773 . 16723802

[15] Wiklund U, Akay M, Morrison S, Niklasson U. Wavelet decomposition of cardiovascular signals for baroreceptor function tests in pigs. IEEE Trans Biomed Eng. 2002;49(7):651–61. doi: 10.1109/TBME.2002.1010848 . 12083299

[16] Voora D, Ginsburg GS. Clinical application of cardiovascular pharmacogenetics. J Am Coll Cardiol. 2012;60(1):9–20. doi: 10.1016/j.jacc.2012.01.067 . 22742397

[17] Koopmans AB, Braakman MH, Vinkers DJ, Hoek HW, van Harten PN. Meta-analysis of probability estimates of worldwide variation of CYP2D6 and CYP2C19. Transl Psychiatry. 2021;11(1):141. Epub 20210224. doi: 10.1038/s41398-020-01129-1 ; PubMed Central PMCID: PMC7904867. 33627619 PMC7904867

[18] Tipton MJ, Kelleher PC, Golden FS. Supraventricular arrhythmias following breath-hold submersions in cold water. Undersea Hyperb Med. 1994;21(3):305–13. . 7950804

[19] Asplund CA, Creswell LL. Hypothesised mechanisms of swimming-related death: a systematic review. Br J Sports Med. 2016;50(22):1360–6. Epub 2016/11/03. doi: 10.1136/bjsports-2015-094722 . 26941276

[20] Ishikawa H, Matsushima M, Nagashima M, Osuga A. Screening of children with arrhythmias for arrhythmia development during diving and swimming—face immersion as a substitute for diving and exercise stress testing as a substitute for swimming. Jpn Circ J. 1992;56(9):881–90. Epub 1992/09/01. doi: 10.1253/jcj.56.881 . 1404843

[21] Moss AJ, Robinson JL, Gessman L, Gillespie R, Zareba W, Schwartz PJ, et al. Comparison of clinical and genetic variables of cardiac events associated with loud noise versus swimming among subjects with the long QT syndrome. Am J Cardiol. 1999;84(8):876–9. Epub 1999/10/26. doi: 10.1016/s0002-9149(99)00458-0 . 10532503

[22] Zeppenfeld K, Tfelt-Hansen J, de Riva M, Winkel BG, Behr ER, Blom NA, et al. 2022 ESC Guidelines for the management of patients with ventricular arrhythmias and the prevention of sudden cardiac death. Eur Heart J. 2022;43(40):3997–4126. doi: 10.1093/eurheartj/ehac262 . 36017572

[23] Priori SG, Blomström-Lundqvist C, Mazzanti A, Blom N, Borggrefe M, Camm J, et al. 2015 ESC Guidelines for the management of patients with ventricular arrhythmias and the prevention of sudden cardiac death: The Task Force for the Management of Patients with Ventricular Arrhythmias and the Prevention of Sudden Cardiac Death of the European Society of Cardiology (ESC). Endorsed by: Association for European Paediatric and Congenital Cardiology (AEPC). Eur Heart J. 2015;36(41):2793–867. Epub 20150829. doi: 10.1093/eurheartj/ehv316 . 26320108

[24] Choi G, Kopplin LJ, Tester DJ, Will ML, Haglund CM, Ackerman MJ. Spectrum and frequency of cardiac channel defects in swimming-triggered arrhythmia syndromes. Circulation. 2004;110(15):2119–24. Epub 2004/10/07. doi: 10.1161/01.CIR.0000144471.98080.CA . 15466642

[25] Batra AS, Silka MJ. Mechanism of sudden cardiac arrest while swimming in a child with the prolonged QT syndrome. J Pediatr. 2002;141(2):283–4. Epub 2002/08/17. doi: 10.1067/mpd.2002.126924 . 12183730

[26] Muneuchi J, Sugitani Y, Watanabe M. Inducible torsades de pointes during an acute face immersion test in an adolescent with type 2 long QT syndrome. Cardiol Young. 2020;30(8):1171–2. Epub 20200721. doi: 10.1017/S1047951120002176 . 32690115

[27] Shattock MJ, Tipton MJ. ’Autonomic conflict’: a different way to die during cold water immersion? J Physiol. 2012;590(14):3219–30. Epub 2012/05/02. doi: 10.1113/jphysiol.2012.229864 ; PubMed Central PMCID: PMC3459038. 22547634 PMC3459038

[28] Winbo A, Ramanan S, Eugster E, Rydberg A, Jovinge S, Skinner JR, et al. Functional hyperactivity in long QT syndrome type 1 pluripotent stem cell-derived sympathetic neurons. Am J Physiol Heart Circ Physiol. 2021;321(1):H217–h27. Epub 2021/06/19. doi: 10.1152/ajpheart.01002.2020 . 34142889

[29] Yoshinaga M, Kamimura J, Fukushige T, Kusubae R, Shimago A, Nishi J, et al. Face immersion in cold water induces prolongation of the QT interval and T-wave changes in children with nonfamilial long QT syndrome. Am J Cardiol. 1999;83(10):1494–7, a8. Epub 1999/05/21. doi: 10.1016/s0002-9149(99)00131-9 . 10335770

[30] Marstrand P, Almatlouh K, Kanters JK, Graff C, Christensen AH, Bundgaard H, et al. Long QT syndrome type 1 and 2 patients respond differently to arrhythmic triggers: The TriQarr in vivo study. Heart Rhythm. 2021;18(2):241–9. Epub 2020/09/04. doi: 10.1016/j.hrthm.2020.08.017 . 32882399

[31] Cook JR, Bigger JT Jr., Kleiger RE, Fleiss JL, Steinman RC Rolnitzky LM. Effect of atenolol and diltiazem on heart period variability in normal persons. J Am Coll Cardiol. 1991;17(2):480–4. doi: 10.1016/s0735-1097(10)80119-6 . 1991906

[32] Niemelä MJ, Airaksinen KE, Huikuri HV. Effect of beta-blockade on heart rate variability in patients with coronary artery disease. J Am Coll Cardiol. 1994;23(6):1370–7. doi: 10.1016/0735-1097(94)90379-4 . 8176095

[33] Tuininga YS, Crijns HJ, Brouwer J, van den Berg MP, Man in’t Veld AJ, Mulder G, et al. Evaluation of importance of central effects of atenolol and metoprolol measured by heart rate variability during mental performance tasks, physical exercise, and daily life in stable postinfarct patients. Circulation. 1995;92(12):3415–23. doi: 10.1161/01.cir.92.12.3415 . 8521562

[34] Vesalainen RK, Kantola IM, Airaksinen KE, Tahvanainen KU, Kaila TJ. Vagal cardiac activity in essential hypertension: the effects of metoprolol and ramipril. Am J Hypertens. 1998;11(6 Pt 1):649–58. doi: 10.1016/s0895-7061(98)00021-1 . 9657623

[35] Eyre EL, Duncan MJ, Birch SL, Fisher JP. The influence of age and weight status on cardiac autonomic control in healthy children: a review. Auton Neurosci. 2014;186:8–21. Epub 20141002. doi: 10.1016/j.autneu.2014.09.019 . 25458714

[36] Harteveld LM, Nederend I, Ten Harkel ADJ, Schutte NM, de Rooij SR, Vrijkotte TGM, et al. Maturation of the Cardiac Autonomic Nervous System Activity in Children and Adolescents. J Am Heart Assoc. 2021;10(4):e017405. Epub 20210202. doi: 10.1161/JAHA.120.017405 ; PubMed Central PMCID: PMC7955328. 33525889 PMC7955328

[37] Lundström A, Wiklund U, Law L, Jensen S, Karlsson M, Rydberg A. Aberrant autonomic pattern during the post-exercise recovery phase in long QT syndrome patients. Auton Neurosci. 2021;236:102897. Epub 20211015. doi: 10.1016/j.autneu.2021.102897 . 34775217

[38] DeMaria N, Selmi A, Kashtan S, Xia X, Wang M, Zareba W, et al. Autonomic and Cardiac Repolarization Lability in Long QT Syndrome Patients. Auton Neurosci. 2020;229:102723. Epub 20200906. doi: 10.1016/j.autneu.2020.102723 ; PubMed Central PMCID: PMC7704776. 32942226 PMC7704776

[39] Winter J, Tipton MJ, Shattock MJ. Autonomic conflict exacerbates long QT associated ventricular arrhythmias. J Mol Cell Cardiol. 2018;116:145–54. Epub 2018/02/07. doi: 10.1016/j.yjmcc.2018.02.001 ; PubMed Central PMCID: PMC5855091. 29408217 PMC5855091

