# Supplementary material for: Cardiac response to water activities in children with Long QT syndrome type 1
Source: PLoS One. 2023 Dec 7;18(12):e0295431. doi: 10.1371/journal.pone.0295431 (PMC10703314; doi:10.1371/journal.pone.0295431)
Supplement: S1 Checklist — (DOCX) [file pone.0295431.s001.docx]

STROBE Statement—checklist of items that should be included in reports of observational studies

|  | Item No. | Recommendation | Page  No. | Relevant text from manuscript |
| --- | --- | --- | --- | --- |
| **Title and abstract** | 1 | (*a*) Indicate the study’s design with a commonly used term in the title or the abstract | 3 | In this cross-sectional study |
|  |  | (*b*) Provide in the abstract an informative and balanced summary of what was done and what was found | 3 | A significantly lower heart rate decrease in response to water activities was seen in LQT1 subjects compared to healthy controls. The data suggests an impaired parasympathetic response in LQT1 children and adolescents. |
| Introduction | | | |  |
| Background/rationale | 2 | Explain the scientific background and rationale for the investigation being reported | 5 | Swimming and enjoying water activities is central to many families. However, in LQT1 the risk of triggering symptoms is particularly increased during these activities, and further exacerbated during adolescence. As there are few studies on cardiac response during water activities in children and adolescents with LQTS, this constitutes a clear knowledge gap. |
| Objectives | 3 | State specific objectives, including any prespecified hypotheses | 6 | The aim of this study was to assess the presence of arrhythmias and to examine the autonomic response to water activities in children and adolescents with LQT1 and compare it with healthy controls. |
| Methods | | | |  |
| Study design | 4 | Present key elements of study design early in the paper | 6-7 | In the Abstract and method section the study design is presented. |
| Setting | 5 | Describe the setting, locations, and relevant dates, including periods of recruitment, exposure, follow-up, and data collection | 6-7 | Recruitment and data collection occurred during the years 2019-2022. |
| Participants | 6 | (*a*) *Cohort study*—Give the eligibility criteria, and the sources and methods of selection of participants. Describe methods of follow-up  *Case-control study*—Give the eligibility criteria, and the sources and methods of case ascertainment and control selection. Give the rationale for the choice of cases and controls  *Cross-sectional study*—Give the eligibility criteria, and the sources and methods of selection of participants | 6 | Genetically verified LQT1 patients between 6 and 19 years old, who attended regular cardiology follow-up at the Department of Pediatric Cardiology, Umeå University Hospital, Sweden. One-on-one age- and sex-matched healthy controls. |
|  |  | (*b*) *Cohort study*—For matched studies, give matching criteria and number of exposed and unexposed  *Case-control study*—For matched studies, give matching criteria and the number of controls per case |  |  |
| Variables | 7 | Clearly define all outcomes, exposures, predictors, potential confounders, and effect modifiers. Give diagnostic criteria, if applicable | 8 | The presence of arrhythmic beats was noted. Heart rate variability (HRV) was analyzed during FI and WBS for subjects with a duration of >10 seconds for each event. |
| Data sources/ measurement | 8* | For each variable of interest, give sources of data and details of methods of assessment (measurement). Describe comparability of assessment methods if there is more than one group | 7 | The Actiwave-Cardio monitor (CamNtech, Cambridge, UK) was used to continuously record the ECG |
| Bias | 9 | Describe any efforts to address potential sources of bias | 6 | One-on-one age- and sex-matched healthy controls. |
| Study size | 10 | Explain how the study size was arrived at | Separately up-loaded | Figure 1 |

Continued on next page

| Quantitative variables | 11 | Explain how quantitative variables were handled in the analyses. If applicable, describe which groupings were chosen and why | 8-9 | QTc in the LQT1 patients was manually calculated from a standard 12-lead resting ECG and analysed as median ± standard deviation. The heart rate response was determined by calculating group averages and standard error of the mean. HRV data were analyzed in the different power spectra (total, LF, HF,LF/HF) as these different spectra mirror different parts of the autonomic nervous system . |
| --- | --- | --- | --- | --- |
| Statistical methods | 12 | (*a*) Describe all statistical methods, including those used to control for confounding | 9-10 | The overall heart rate response was determined by calculating group averages and standard error of the mean (SEM) of the equidistantly sampled data, where the responses were smoothed by calculating one-second moving averages. QTc was reported as median ± standard deviation (SD). Comparisons between the two groups regarding heart rate response, duration of the events and QTc were conducted with two-sampled t-tests. HRV data were analyzed by analysis of variance (ANOVA) for repeated measurements with time, group, and their interactions as variables. A p-value of less than 0.05 was considered statistically significant. |
|  |  | (*b*) Describe any methods used to examine subgroups and interactions |  | The study group was too small, to allow any sub-analysis. |
|  |  | (*c*) Explain how missing data were addressed |  | NA |
|  |  | (*d*) *Cohort study*—If applicable, explain how loss to follow-up was addressed  *Case-control study*—If applicable, explain how matching of cases and controls was addressed  *Cross-sectional study*—If applicable, describe analytical methods taking account of sampling strategy |  | NA |
|  |  | (*e*) Describe any sensitivity analyses |  | NA |
| Results | | | | |
| Participants | 13* | (a) Report numbers of individuals at each stage of study—eg numbers potentially eligible, examined for eligibility, confirmed eligible, included in the study, completing follow-up, and analysed | Separately up-loaded | Figure 1 |
|  |  | (b) Give reasons for non-participation at each stage | 10 | Two declined to participate (Figure 1). |
|  |  | (c) Consider use of a flow diagram | Separately up-loaded | Figure 1 |
| Descriptive data | 14* | (a) Give characteristics of study participants (eg demographic, clinical, social) and information on exposures and potential confounders | 10-11 | Table 1 |
|  |  | (b) Indicate number of participants with missing data for each variable of interest | 10 | Of the 15 matched LQT1 patient and control pairs, 12 had a duration time >10 seconds for events 1 and 2 (FI and WBS) and were included in the heart rate and HRV analysis. |
|  |  | (c) *Cohort study*—Summarise follow-up time (eg, average and total amount) |  |  |
| Outcome data | 15* | *Cohort study*—Report numbers of outcome events or summary measures over time |  |  |
|  |  | *Case-control study—*Report numbers in each exposure category, or summary measures of exposure |  |  |
|  |  | *Cross-sectional study—*Report numbers of outcome events or summary measures | 11-14 | See RESULTS |
| Main results | 16 | (*a*) Give unadjusted estimates and, if applicable, confounder-adjusted estimates and their precision (eg, 95% confidence interval). Make clear which confounders were adjusted for and why they were included | 11-14 | Table 2 and table 3 |
|  |  | (*b*) Report category boundaries when continuous variables were categorized | 8-9 | In the method section continuous variables were categorized in different power spectra and category boundaries were reported. |
|  |  | (*c*) If relevant, consider translating estimates of relative risk into absolute risk for a meaningful time period |  | NA |

Continued on next page

| Other analyses | 17 | Report other analyses done—eg analyses of subgroups and interactions, and sensitivity analyses |  | NA |
| --- | --- | --- | --- | --- |
| Discussion | | | | |
| Key results | 18 | Summarise key results with reference to study objectives | 15 | We have assessed the presence of arrhythmias and the autonomic response to water activities in children and adolescents with LQT1 while on their regular betablocker therapy. None of the included LQT1 patients had malignant arrhythmias during the water activities. However, a less pronounced heart rate decrease was seen in LQT1 patients, compared to healthy age- and sex-matched controls. Furthermore, during face immersion and whole-body submersion, the LQT1 patients had a decreased HRV, especially in the high frequency spectrum, corresponding to parasympathetic activity. These findings indicate differences between LQT1 patients and controls in autonomic response and suggest that the diving reflex provoked a less pronounced parasympathetic activation in the LQT1 patients. |
| Limitations | 19 | Discuss limitations of the study, taking into account sources of potential bias or imprecision. Discuss both direction and magnitude of any potential bias | 19-20 | All our LQT1 patients were on betablocker therapy, as therapy discontinuation was not feasible from an ethical point of view. This limited our ability to assess the sympathetic response to water activities. Moreover, due to the short duration of recordings, the slower sympathetic response was potentially not captured in full, and not possible to evaluate in isolation as LF represents both sympathetic and parasympathetic activity.  However, in another study of adults, betablocker therapy had little influence on the heart rate reduction during simulated diving. Even though it was not significant, the tendency was rather that the heart reduction was greater under the influence of betablocker therapy. When comparing these results from 10 seconds of face immersion, with our patient’s HR after 10 seconds, a similar pattern can be seen (Suppl. Figure A.2.). |
| Interpretation | 20 | Give a cautious overall interpretation of results considering objectives, limitations, multiplicity of analyses, results from similar studies, and other relevant evidence | 20 | The results in this study indicate an impaired parasympathetic reaction in response to face immersion and whole-body submersion in LQTS type 1 children compared with healthy controls. This aberrant ANS response may cause an autonomic imbalance, and our findings are in accordance with previous studies that demonstrate a deviant parasympathetic response. Furthermore, these results provide insight that alterations in the autonomic tone may contribute to the arrhythmogenesis in LQTS and therefore emphasizes the importance of studying both branches of the autonomic nervous system. |
| Generalisability | 21 | Discuss the generalisability (external validity) of the study results | 20 | Despite the small size of the study, it is an interesting contribution to the increasing understanding of arrhythmia formation in Long QT Syndrome. |
| Other information | |  | | |
| Funding | 22 | Give the source of funding and the role of the funders for the present study and, if applicable, for the original study on which the present article is based | 20 | This work was supported by the Swedish Heart Lung Foundation and through a regional agreement between Umeå University and Region Västerbotten (ALF). |

*Give information separately for cases and controls in case-control studies and, if applicable, for exposed and unexposed groups in cohort and cross-sectional studies.

**Note:** An Explanation and Elaboration article discusses each checklist item and gives methodological background and published examples of transparent reporting. The STROBE checklist is best used in conjunction with this article (freely available on the Web sites of PLoS Medicine at http://www.plosmedicine.org/, Annals of Internal Medicine at http://www.annals.org/, and Epidemiology at http://www.epidem.com/). Information on the STROBE Initiative is available at www.strobe-statement.org.
