# Supplementary material for: Cardiac response to water activities in children with Long QT syndrome type 1
Source: PLoS One. 2023 Dec 7;18(12):e0295431. doi: 10.1371/journal.pone.0295431 (PMC10703314; doi:10.1371/journal.pone.0295431)
Supplement: S1 Table — (DOCX) [file pone.0295431.s002.docx]

# Supplementary to the article: Cardiac response to water activities in children with Long QT syndrome type 1

## Anna Lundström, MD^a^, Urban Wiklund, MSc, PhD^b^, Annika Winbo, MD, PhD^a,c^, Håkan Eliasson, MD, PhD^d^, Marcus Karlsson^b^, Annika Rydberg, MD, PhD^a^

a Department of Clinical Sciences, Pediatrics, Umeå University, 901 85, Umeå, Sweden.

b Department of Radiation Sciences, Biomedical Engineering, Umeå University, 901 87, Umeå, Sweden.

## c Department of Physiology, University of Auckland, Auckland, Private Bag 92019, 1023 New Zealand.

## d Department of Women's and Children's Health, Karolinska Institute, Widerströmska huset, Tomtebodavägen 18A, 171 77, Stockholm, Sweden.

**S1 Table.** **Genetic variants found in the LQTS group.**

| **Gene** | **cDNA change** | **Protein change** | **Nb of patients** | **ACMG classification** |
| --- | --- | --- | --- | --- |
| KCNQ1 | c.332A>G | p.Tyr111Cys | 13 | Pathogenic |
| KCNQ1 | c.1552C>T | p.Arg518* | 1 | Pathogenic |
| KCNQ1 | c.1588C>T | p.Gln530* | 1 | Pathogenic |

ACMG = American College of Medical Genetics and Genomics [1].

**References:**

1. Richards S, Aziz N, Bale S, Bick D, Das S, Gastier-Foster J, et al. Standards and guidelines for the interpretation of sequence variants: a joint consensus recommendation of the American College of Medical Genetics and Genomics and the Association for Molecular Pathology. Genet Med. 2015;17(5):405-24. Epub 20150305. doi: 10.1038/gim.2015.30. PubMed PMID: 25741868; PubMed Central PMCID: PMCPMC4544753.
