# Supplementary material for: Cardiac response to water activities in children with Long QT syndrome type 1
Source: PLoS One. 2023 Dec 7;18(12):e0295431. doi: 10.1371/journal.pone.0295431 (PMC10703314; doi:10.1371/journal.pone.0295431)
Supplement: S2 Table — (DOCX) [file pone.0295431.s003.docx]

# Supplementary to the article: Cardiac response to water activities in children with Long QT syndrome type 1

## Anna Lundström, MD^a^, Urban Wiklund, MSc, PhD^b^, Annika Winbo, MD, PhD^a,c^, Håkan Eliasson, MD, PhD^d^, Marcus Karlsson^b^, Annika Rydberg, MD, PhD^a^

a Department of Clinical Sciences, Pediatrics, Umeå University, 901 85, Umeå, Sweden.

b Department of Radiation Sciences, Biomedical Engineering, Umeå University, 901 87, Umeå, Sweden.

## c Department of Physiology, University of Auckland, Auckland, Private Bag 92019, 1023 New Zealand.

## d Department of Women's and Children's Health, Karolinska Institute, Widerströmska huset, Tomtebodavägen 18A, 171 77, Stockholm, Sweden.

**S2 Table.** **Swimming protocol**.


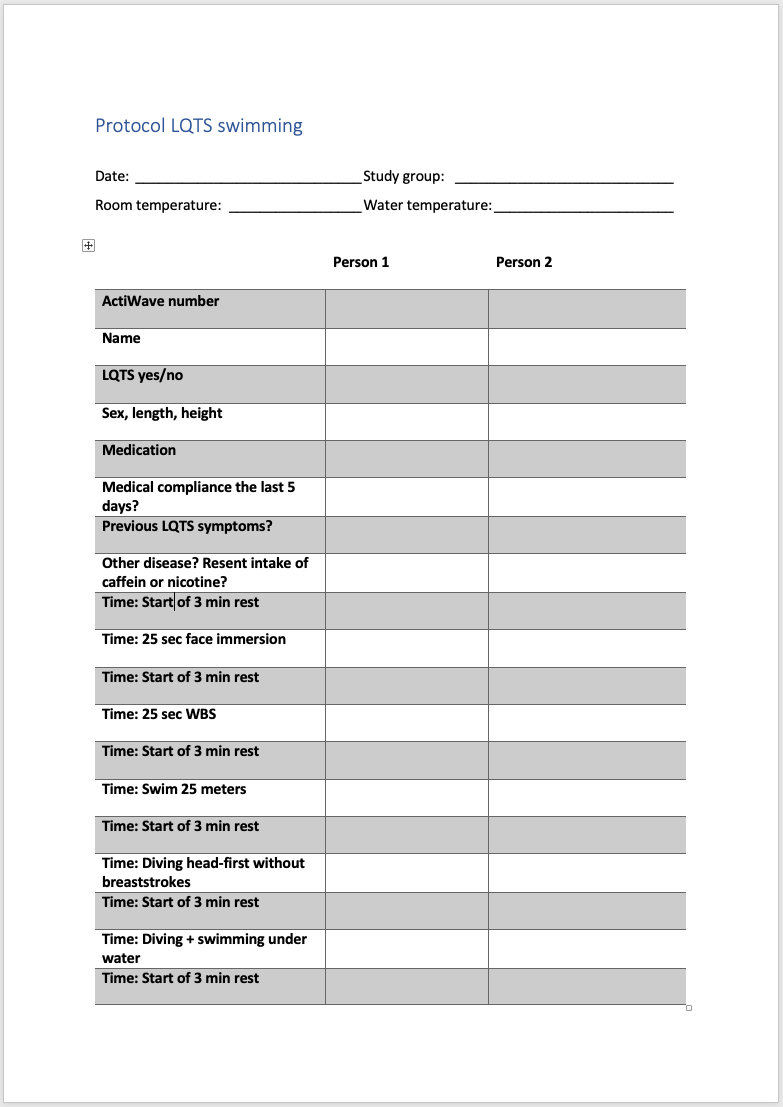


LQTS = Long QT syndrome, WBS = whole-body submersion
